# The morphology of permanent maxillary first molars evaluated by cone-beam computed tomography among a Yemeni population

**DOI:** 10.1186/s12903-023-02752-2

**Published:** 2023-01-27

**Authors:** Abdulbaset A. Mufadhal, Ahmed A. Madfa

**Affiliations:** 1 Department of Conservative Dentistry and Endodontics, Faculty of Dentistry, Sana’a University, Sana’a, Yemen; 2 Department of Restorative Dental Science, Collage of Dentistry, University of Ha’il, Ha’il, Kingdom of Saudi Arabia; 3 Department of Conservative Dentistry, Faculty of Dentistry, Thamar University, Dhamar, Yemen

**Keywords:** Cone beam computed tomography, Endodontics, Maxillary first molar, Root canal morphology, Yemeni population

## Abstract

**Background:**

The study's objective was to use CBCT to ascertain the root and root canal anatomy of the permanent maxillary first molars in a Yemeni population. It was considered how gender affected the prevalence of root canal morphology.

**Methods:**

A sample of 373 CBCT records of maxillary permanent first molars belonging to 373 Yemeni individuals (162 males and 211 females) aged between 12 and 65 years were included in this study. Using CBCT on the teeth, the root form and canal morphology for each root based on Vertucci's classification were evaluated. The distribution of MB2 occurrence was documented. The prevalence and resemblance of the men and females were investigated. The Chi-square test was performed to evaluate the findings.

**Results:**

Of the 373 maxillary first molars studied, the three separated root form was found in 94.9% of the studied MFMs while the two fused and one separate root form was found in 4.5%. The majority of the three-rooted MFMs (82.6%) had more than three root canals (four canals in 77.2% and five canals in 5.4%). The prevalence of MB2 was as high as 82.3% of the three-rooted MFMs. Vertucci type II configuration was the most frequent canal type (25%) followed by Vertucci type III (23.1%) and Vertucci type I (17.7%) in the mesiobuccal root. Vertucci type I was the most prevalent canal configuration in the distobuccal root and palatal root of the three-rooted MFMs (96% and 99.5%, respectively).

**Conclusions:**

The maxillary first molars' root canal morphology indicated notable variations among a Yemeni population. The maxillary first molars of most of the Yemeni participants in this study had three roots and four canals. In light of the high occurrence of MB2 (82.3%) in permanent maxillary first molars, our findings emphasize the need of searching for and using cutting-edge techniques to locate the MB2 canals. Males outnumbered females in proportion.

## Background

The entire root canal system must be thoroughly cleaned, shaped, and three-dimensionally sealed for root canal therapy to be successful. To accomplish these goals, a thorough understanding of root and canal anatomy is necessary [1]. A lack of such information raises the possibility of missed root canals and procedural errors, which may cause the therapy to fail in the end [2]. The anatomy of the root canal system, unfortunately, exhibits tremendous complexity and considerable variability [3], which increases the requirement for dental professionals to be conversant with such differences.

Since root canal anatomy is genetically driven, distinct groups' patterns of root canal morphology may resemble one another as well as differ from one another. Numerous studies [4–6] have found root canal anatomical variations related to ethnicity. To better inform clinicians on population trends in the anatomy of the root canal system, it would be beneficial to characterize the root canal morphology of a particular community and compare the results with those of other populations. Therefore, it's important to assess differences in root and canal shape among various racial populations and subpopulations.

The maxillary first molar (MFM) is one of the first permanent teeth to erupt in the oral cavity, making it more vulnerable to decay and root canal therapy. However, the maxillary molars' root canal morphology is one of the most intricate root canal anatomies found in human teeth, making it difficult to treat with endodontics. For instance, during root canal therapy, the second canals in the mesiobuccal root are the ones that are most frequently overlooked [7]. As a result, numerous investigations have been carried out globally [8–12] to assess the root and canal morphology of the permanent MFM.

The root form and canal configuration of MFMs in various groups have been studied in numerous anatomical studies [11–17]. Several anatomical variants relating to the number and morphology of their roots and root canals have been observed, even though the most typical form of MFMs includes three separated roots and four canals [4]. There have been reports of various proportions of one-rooted, two-rooted, and four-rooted MFMs in various populations [13, 18–20]. However, the MFMs have not been investigated in the Yemeni population which is predominantly composed of Arab ethnicity with very small minority groups of Horn Africans and South Asians. Considering this, the study's objective was to use CBCT to ascertain the root and root canal anatomy of the permanent MFMs in a Yemeni population. It was taken into account how gender affected the prevalence of root canal morphology.

## Method

### Study design and bioethical considerations

A retrospective cross-sectional observational study was conducted to determine the root form and canal morphology of the permanent MFMs in a Yemeni population using CBCT. Formally, an ethics approval (No. 1075) was achieved from the Medical Ethics Committee in the Faculty of Dentistry at Sana’a University. Due to the study's retroactive character, the ethical Committee of the College of Dentistry, Sana’a University, waived informed consent. To maintain patients’ confidentiality, only their gender and age were recorded, and the rest of their demographic data were omitted. All collected information was managed confidentially, and the study findings will hopefully represent a reference for an endodontist and dental practitioners in their clinical practice. CBCT images of MFMs were collected from patients who had undergone CBCT scanning for diagnostic purposes.

### Study population

Patients attending two imaging centers in Sana'a city were considered as the study population for the current study. These include Alwaleed 3D Green X-Ray Center and Saba 3D Green Scan Center which are generally considered one of the most prominent head and neck imaging centers in the city. These centers are geographically located in two different and far distant areas where dental clinics and centers are more concentrated than any other regions in the city and to which many patients from Sana’a city and surrounding areas attend to get oral and dental care. In General, Sana’a city contains a mixed population of more than three million from different governorates of the country.

A non-probability purposive sampling method was used in this study. A database of 2000 CBCT scans was evaluated and a sample of 373 CBCT records of maxillary permanent first molars belonging to 373 Yemeni individuals (162 males and 211 females) aged between 12 and 65 years were included in the study. The inclusion criteria were non-distorted CBCT scans showing MFMs with fully formed roots in patients aged 12–65 years. Images of teeth treated endodontically or postcoronal restorations or with full-coverage restorations or metallic restorations or creating artifacts in the scans were excluded. Teeth with root resorption or calcification or teeth associated with periapical lesions and low-quality CBCT images were also excluded. The final sample size in this study was 373 CBCT images after examination of the 2000 images according to the inclusion/exclusion criteria.

### CBCT image acquisition parameters

The CBCT imaging in both centers was performed by using the PaX-i3D Green imaging machine (VATECH Co., Ltd., Gyeonggi-do, Korea) following the recommended protocol of the manufacturer. The acquisition parameters were as follows: 50–99 Kvp, 4–16 mA, 7.2–12 s exposure time, fields of view (FOV) of 5 × 5, 8 × 5, and 8 × 8 cm, isotropic Voxel Size of 0.08–0.20 mm and minimum slice thickness of 0.1 mm.

### Processing of the images

The CBCT images were managed using Ez3D-i software (version Ewoosoft, Gyeonggi-do, Korea) and processed in a 64-bit Windows 10 system. All the images were visualized through HP OMEN UHD Graphics 15 inches' screen with a resolution of 1920 × 1080 pixels in a dark room. The contrast, sharpness, and brightness of images were adjusted using the software’s image processing tool to ensure optimal visualization. The teeth were analyzed in all three planes independently (axial, coronal, and sagittal planes).

Using CBCT, the teeth related were inspected for the following observations that estimated:

- The number and morphology of roots
- The number of canals in each root
- Distribution of MB2 occurrence
- The canal morphology for each root according to Vertucci's classification
- Any other additional types.

### Examiners calibration and agreement

Before evaluation, the examiner participated in calibration training. Twenty percent of the sample was selected randomly and evaluated by the examiner and an endodontist (with more than 5 years of endodontic experience). The inter-observer agreement was then determined by calculating the kappa value which was 87.7%. Cases of disagreement were evaluated and discussed by the observers concurrently until a final agreement was reached. Two weeks after the evaluation, a second analysis was performed blindly by the same examiner using approximately 20% of the sample to assess the intra-observer reliability. The difference between the first and the second observations was statistically non-significant.

### Statistical analysis

Data were analyzed using the Statistical Package for the Social Sciences (IBM Co., New York, NY, USA), including frequency distribution and cross-tabulation. The inter-observer agreement was assessed by using the Kappa test. While the intra-observer reliability was checked by using Wilcoxon signed-rank test. The total number of roots and the root canal configuration were analyzed. The chi-squared and Fisher's exact test were used to determine the statistical significance between different parameters. The level of significance was set at 5% (*P* < 0.05).

## Results

### Number and type of roots

**Fig. 1:**
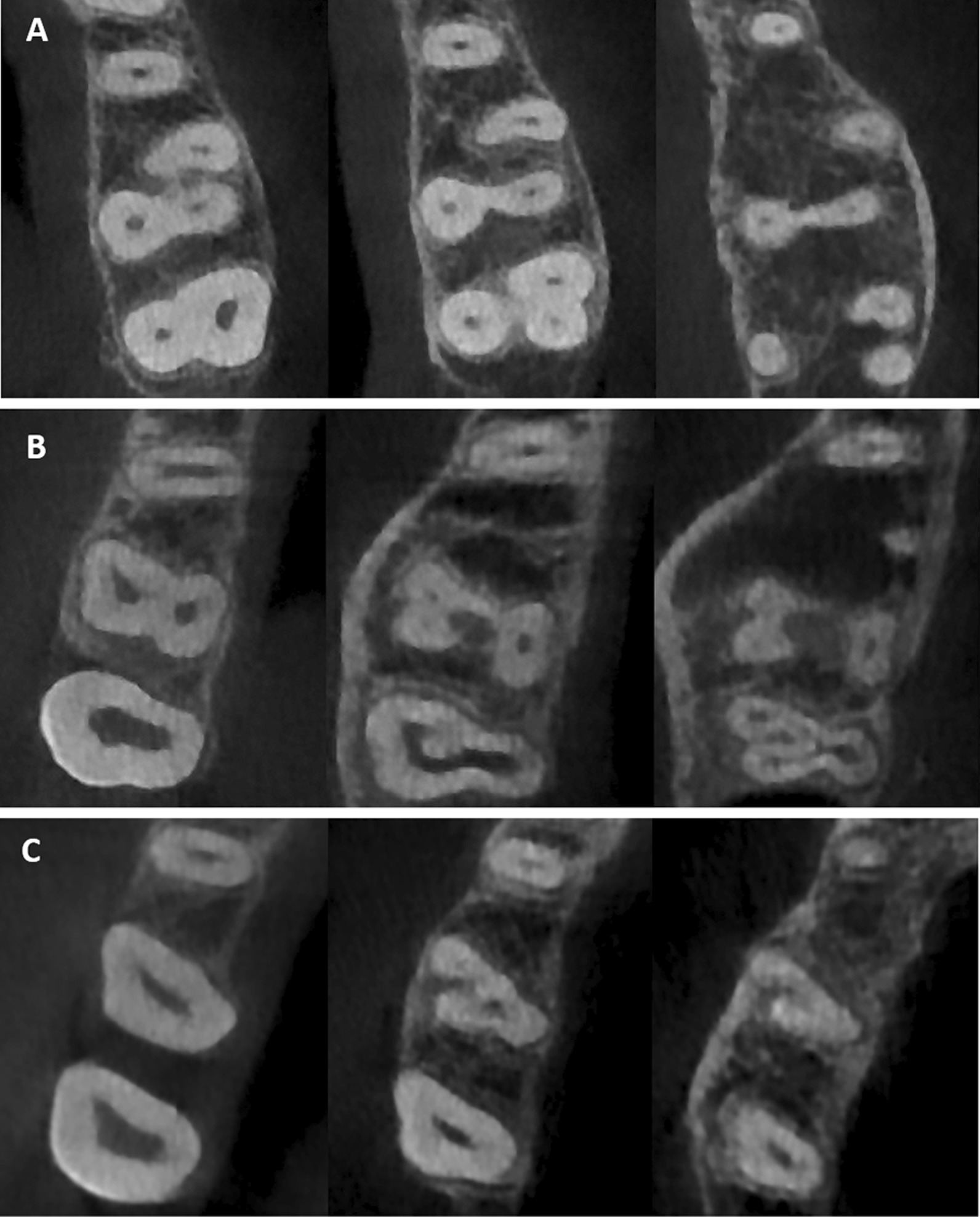
Maxillary first molars with **A** the palatal roots fused to the DB root; **B** the MB root fused to the DB root; **C** the Palatal root fused to both MB & DB roots

Table 1 summarizes the distribution of roots number and types of MFMs among males and females. The three separated root form was found in 94.9% of the studied MFMs while the two fused and one separate root form was found in 4.5%. Only one MFM had three fused roots (Fig. 1). Similarly, the two separated root form was encountered only in one MFM (Fig. 1). There were no significant gender differences regarding the number of roots.

**Table 1:** Number and type of roots among males and females

Number of roots	Male		Female		Total	
	n	%	n	%	n	%
2S	0	0.0	1	0.5	1	0.3
3S	156	96.3	198	93.8	354	94.9
*2F1S*						
Type 1 (Fusion of MBR & DBR)	0	0.0	3	1.4	3	0.8
Type 3 (Fusion of PR & DBR)	5	3.1	9	4.3	14	3.8
*3F*						
Type 5 (Fusion of PR with both MBR & DBR)	1	0.6	0	0.0	1	0.3
X^2^	*P* = 0.26					

2S, two separate roots; 3S, three separate roots; 2F1S, two fused roots and one separate root; 3F, three fused roots

The prevalence of complete fusion (from the CEJ to the apex) of two or more roots of the observed MFMs was equal to 4.8% without gender predilection. Type 3 root fusion (fusion of PR and DBR) was the most prevalent pattern (3.8%) (Fig. 1).

### Number of root canals

**Fig. 2:**
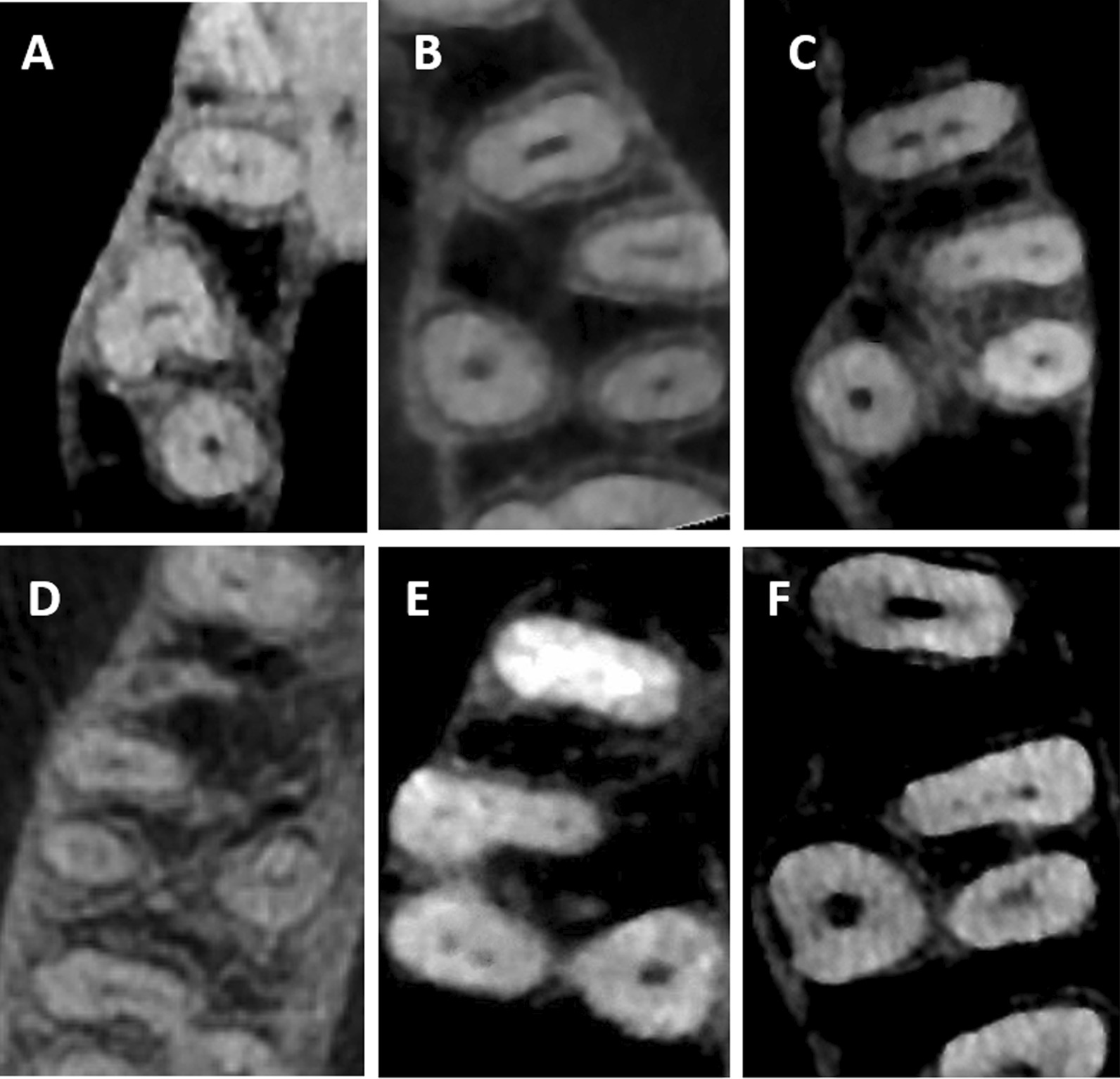
Maxillary first molars with different number of root canals; **A** two root canals (one buccal and one palatal); **B** three root canals (one canal in each root); **C** four root canals (two in the MB root); **D** four root canals (two in the DB root); **E** five root canals (two in the MB root, two in the DB root, and one in the palatal root); **F** five root canals (three in the MB root, one in the DB root, and one in the palatal root)

Table 2 and Fig. 2 show the number of root canals in MFMs among males and females. The majority of the three-rooted MFMs (82.6%) had more than three root canals (four canals in 77.2% and five canals in 5.4%). However, only 17.2% of the studied MFMs had three root canals. Two root canals were found in the two-rooted form MFM. The presence of four and five canals in MFMs among males (79.0% and 6.8% respectively) was higher than those among females (75.8% and 4.3% respectively). However, these differences were statistically not significant.

**Table 2:** Distribution of root canal number among males and females

Number of root canals	Male		Female		Total	
	n	%	n	%	n	%
Two Canals	0	0	1	0.5	1	0.3
Three Canals	23	14.2	41	19.4	64	17.2
*Four Canals*						
2MB, 1DB, 1P	128	79	158	74.8	286	76.6
1MB, 2DB, 1P	0	0	1	0.5	1	0.3
1MB, 1DB, 2P	0	0	1	0.5	1	0.3
Total	128	79	160	75.8	288	77.2
*Five Canals*						
3 MB, 1DB, 1P	4	2.5	1	0.5	5	1.3
2 MB, 2DB, 1P	7	4.3	7	3.3	14	3.8
2 MB, 1DB, 2P	0	0	1	0.5	1	0.3
Total	11	6.8	9	4.3	20	5.4
X^2^	*P* = 0.26

The prevalence of MB2 was as high as 82.3% of the three-rooted MFMs. Moreover, five teeth (1.5%) showed three canals configuration at different levels of their MBR. Table 3 shows that the MB2 canal was found more frequently in males than in females (85.8% and 79.0%, respectively). However, this difference was not statistically significant (*P* = 0.07). In the current study, a second canal in the DBR (DB2) was found in 4% of the MFMs, while a second canal in the PR (P2) was found only in two teeth (0.5%).

**Table 3:** Distribution of MB2 occurrence in MFMs among males and females

Gender	Absence of MB2		Presence of MB2	
	Frequency	(%)	Frequency	(%)
Male	23	(14.2%)	139	(85.8%)
Female	43	(20.5%)	167	(79.5%)
Total	66	(17.7%)	306	(82.3%)
X^2^	*P* = 0.07			

### Root canal configurations

**Fig. 3:**
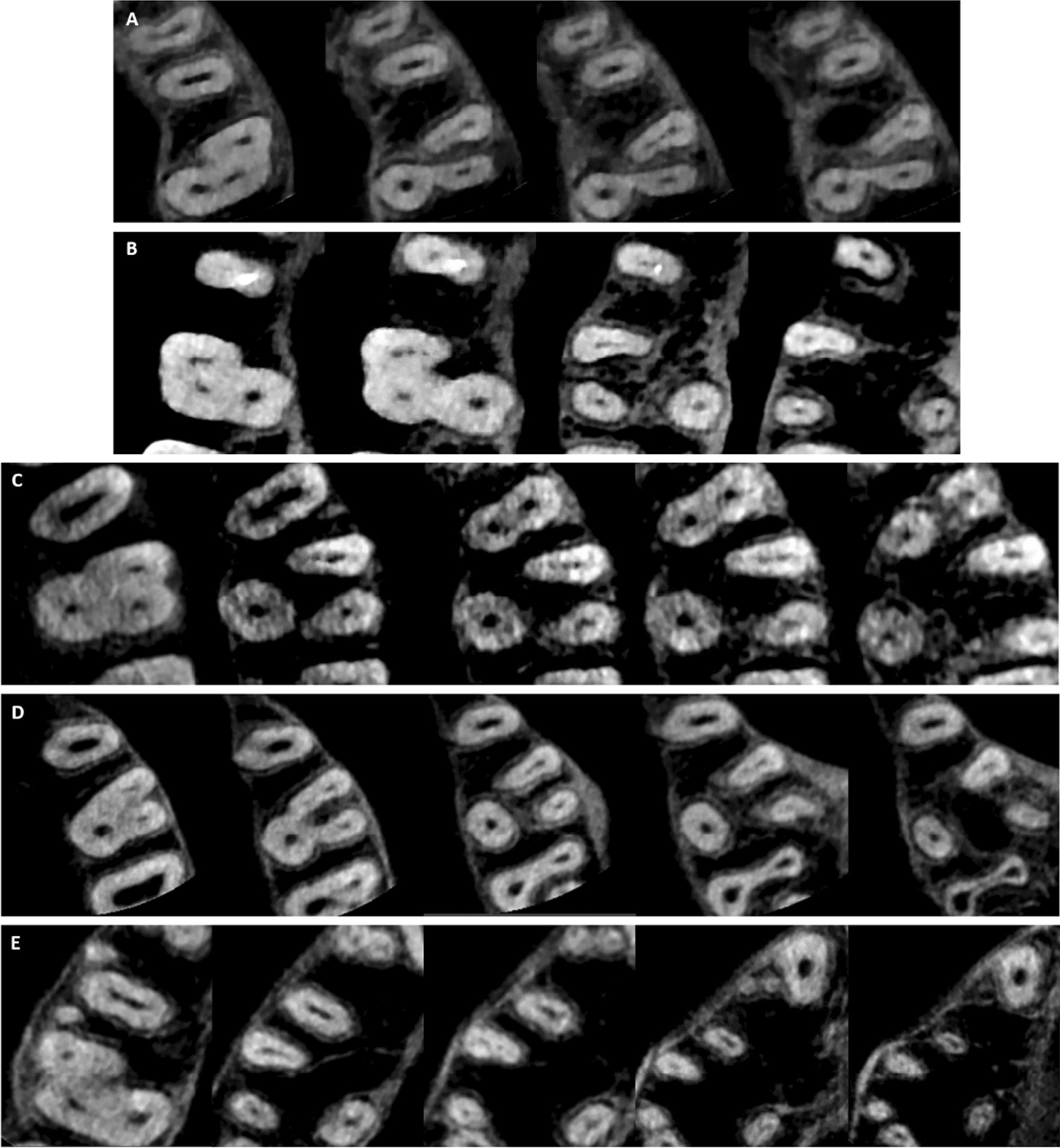
Some of the root canal variations observed in the MB root **A** 1-2-3-2 canal configuration; **B** 1-3-1-2 canal configuration; **C** 1-2-3-2-1 canal configuration; **D** 1-2-1-2-1 canal configuration; **E** 2-1-2-1-2 canal configuration (New root canal type)

**Fig. 4:**
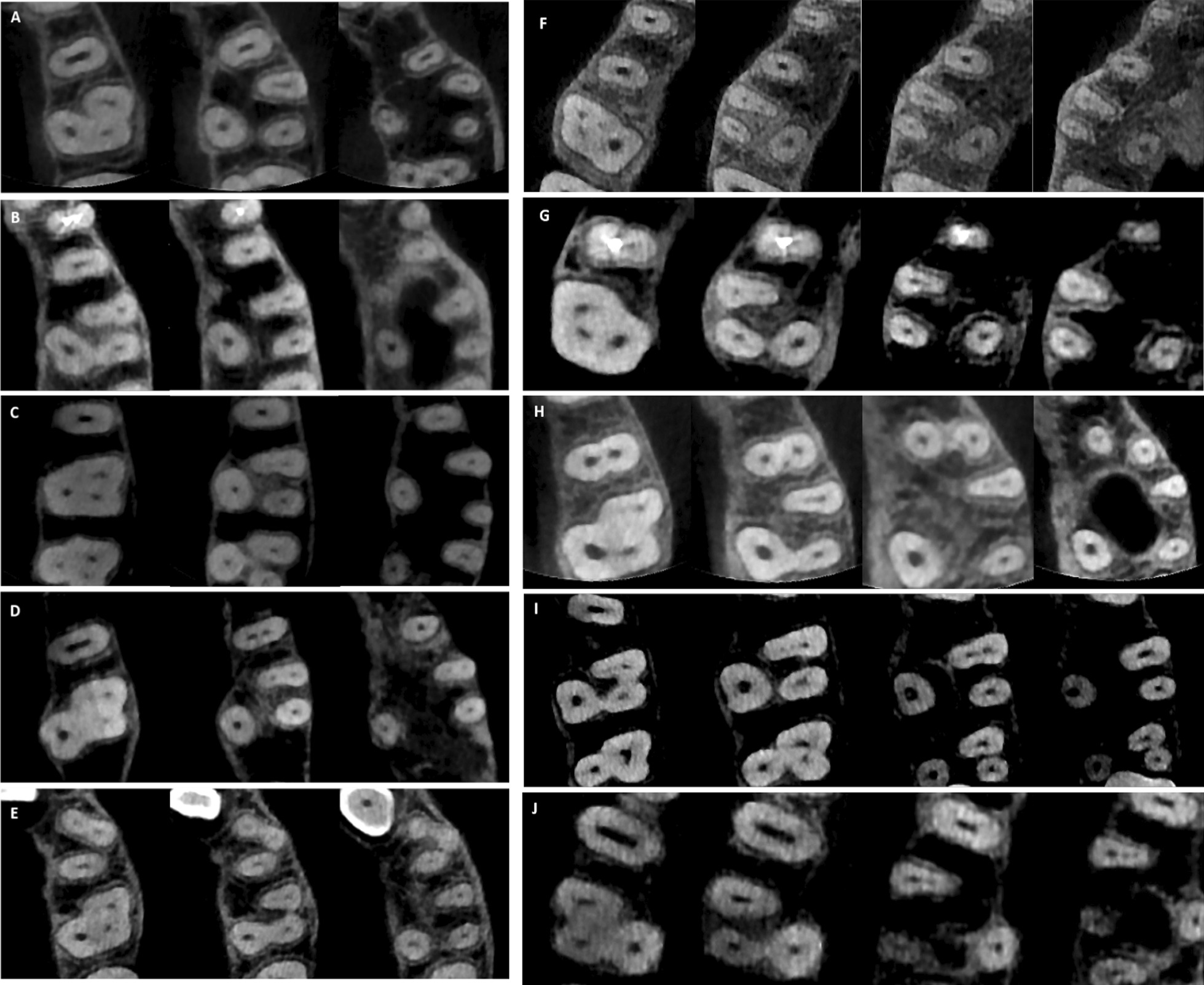
Other Variations of root canal configuration observed in the MB root; **A** Vertucci Type I (1-1); **B** Vertucci Type II (2-1); **C** Vertucci Type III (1-2-1); **D** Vertucci Type IV (2-2); **E** Vertucci Type V (1-2); **F** Vertucci Type VI (2-1-2); **G** Vertucci Type VII (1-2-1-2); **H** (2-1-2-1) canal configuration; I, (3-2-1) canal type; **J** (1-2-3) root canal configuration

**Fig. 5:**
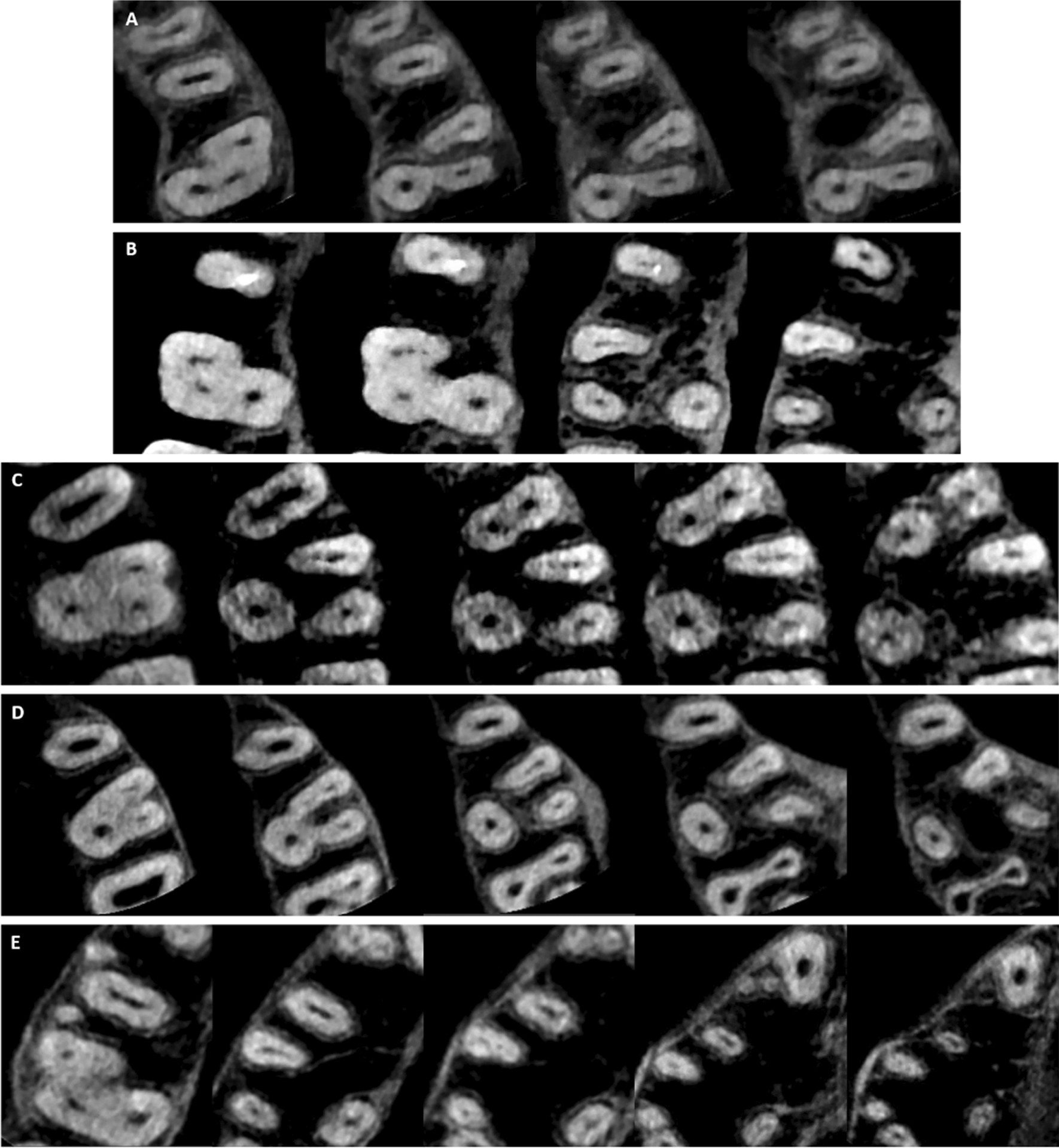
Canal configurations observed in the DB root; **A** Vertucci Type I (1-1); **B** Vertucci Type II (2-1); **C** Vertucci Type III (1-2-1); **D** Vertucci Type V (1-2); **E** Vertucci Type VII (1-2-1-2)

**Fig. 6:**
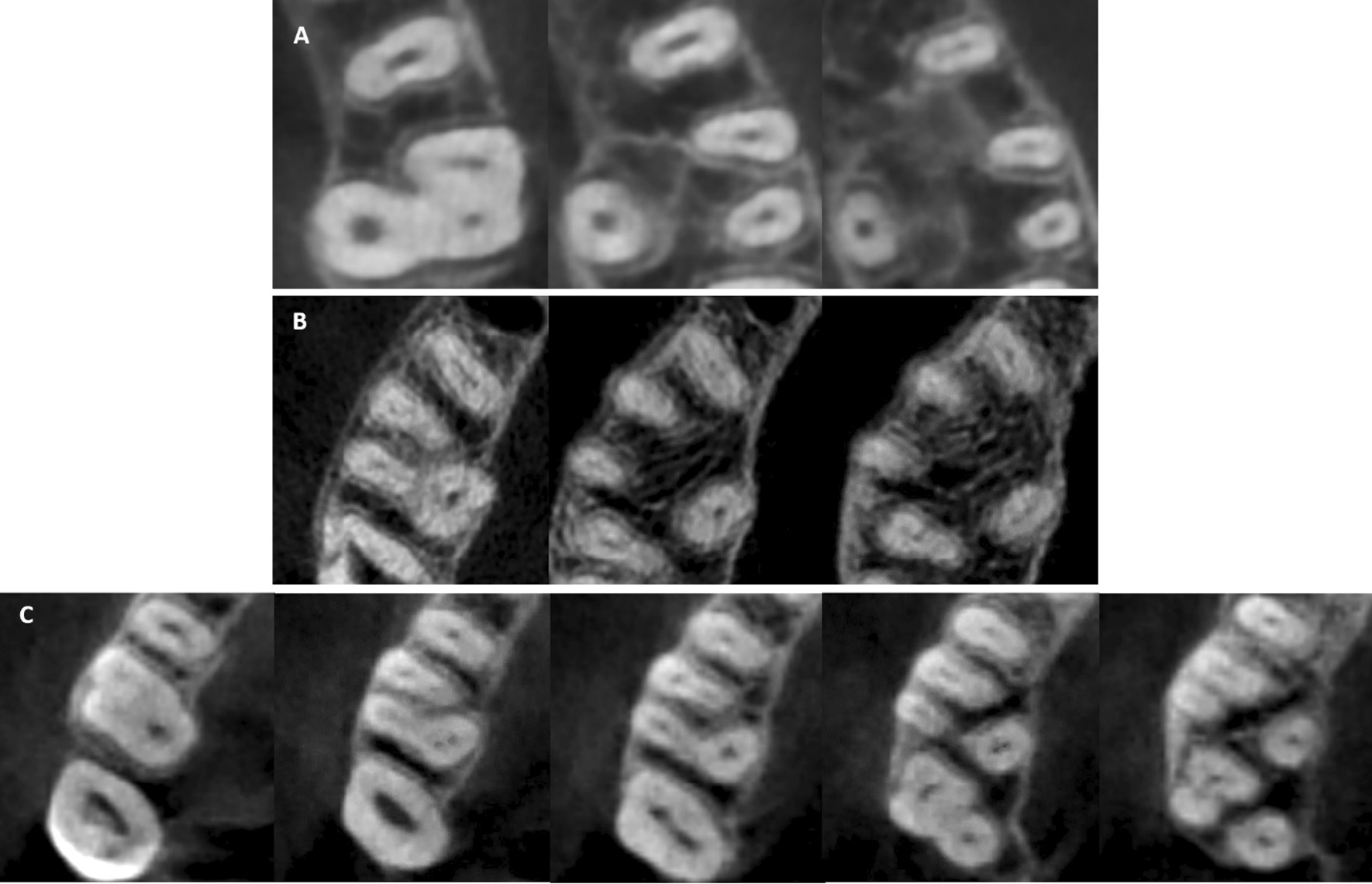
Canal configurations that were observed in the palatal root; **A** Vertucci Type I (1-1); **B** Vertucci Type V (1-2); **C** additional Type (1-2-1-2-1)

Table 4 summarizes the frequency of different root canal configurations in the MFMs. Fifteen different canal types were found in the MBR including the first seven Vertucci types, seven additional canal types that were previously reported, and a new canal type (2-1-2-1-2) that was not reported in earlier published studies (Fig. 3). Vertucci type II configuration was the most frequent canal type (25%) followed by Vertucci type III (23.1%) and Vertucci type I (17.7%) as shown in Fig. 4. Vertucci type I was the most prevalent canal configuration in the DBR and PR of the three-rooted MFMs (96% and 99.5%, respectively) as shown in Figs. 5 and 6. The incidence of root canal configurations of MFMs was not affected by gender (*P* > 0.05).

**Table 4:** Distribution of root canal configurations in three-rooted MFMs according to Vertucci’s classification and other additional types

	Vertucci’s canal types							Additional canal types							New type	Total
	I	II	III	IV	V	VI	VII	2-1-2-1^†^	1-2-3-2^‡^	1-2-3-2-1^‡^	1-3-1-2^‡^	3-2-1^‡^	1-2-1-2-1^¥^	1-2-3^¥^	2-1-2-1-2*	
MBR																
n	66	93	86	42	21	25	8	16	1	1	1	1	8	1	2	372
%	17.7	25	23.1	11.3	5.6	6.7	2.2	4.3	0.3	0.3	0.3	0.3	2.2	0.3	0.5	100
DBR																
n	357	2	11	0	1	0	1	0	0	0	0	0	0	0	0	372
%	96	0.5	3	0	0.3	0	0.3	0	0	0	0	0	0	0	0	100
PR																
n	370	0	0	0	1	0	0	0	0	0	0	0	1	0	0	372
%	99.5	0	0	0	0.3	0	0	0	0	0	0	0	0.3	0	0	100

^†^Gulabivala et al. additional type

^‡^Sert and Bayirli additional types

^¥^Senan et al. additional types

*Not reported in earlier published studies

## Discussion

The anatomy of MFMs in various communities throughout the world has been intensively investigated in recent decades to inspect if there are any ethnic or geographic variances [12–15, 21]. The current study is the first to date to investigate the root form and root canal configuration of MFMs among a Yemeni population.

Several methods have been used for evaluating the root form and canal morphology of human teeth. Although the clearing method has been widely used for many decades and is considered a standard method [1], it is a destructive method that can only be used in vitro for research purposes [22]. In addition, recent studies have reported that the clearing technique shows less fine anatomical details particularly type I canal configuration and more complex anatomical configurations [22, 23]. This limitation might be due to the failure of dye to penetrate and stain the whole root canal system [23]. Recently, more advanced three-dimensional imaging technologies, such as Micro-CT and CBCT, have been introduced to evaluate root canal anatomy [24]. Even though the micro-CT is considered an accurate and reliable method that provides detailed quantitative and qualitative evaluation of root canal anatomy, it is used only in vitro because of its prolonged exposure time and high radiation dose [23]. Furthermore, it is too expensive and not available in developing and under-developed countries. On the other hand, using the CBCT imaging technique for evaluating external and internal dental anatomy is quickly increasing worldwide [25]. It represents a non-destructive reproducible technique for analyzing dental anatomy in a three-dimensional manner. Many studies have reported that CBCT is a non-invasive, accurate, and reliable method for evaluating root canal anatomy when compared to micro-CT or standard clearing methods [12, 23, 26, 27]. Moreover, it can be used in both in vivo and ex vivo situations. Therefore, CBCT was used in this study to evaluate the root and root canal morphology of permanent MFMs among a Yemeni population.

Vertucci canal classification [5] was predominantly used in the current study because of its simplicity and the fact that the majority of authors in the literature use it. Unfortunately, it only proposes eight sorts of canal configurations. Any canal type not covered by this classification was therefore labeled as an extra root canal configuration.

Several anatomical studies in different populations had reported that almost all MFMs presented with three separated roots with very low root fusion prevalence if any. According to Kim et al., roots were considered to be fused if the fusion extended from the CEJ to the apex [17]. The same criteria were considered in the present study. Interestingly, the present study showed that 4.8% of the studied MFMs had a complete fusion of two or more roots. This finding was significantly higher than those reported in Indian, Korean, Saudi, Iranian, and Egyptian populations (0.0%, 0.73%, 0.0%, 1.3%, and 0.0%, respectively) [12–15, 17]. On the other hand, many earlier studies [28, 29], that reported higher root fusion incidence in MFMs (7.7% and 6.2%, respectively), regarded roots to be fused if the fusion occurred along one-third or less of the root surface. Therefore, the criteria for root fusion have to be defined to determine whether such differences are true variations.

The majority (82.6%) of the MFMs in the current study had more than three root canals. These results were comparable with previous studies reported in Iranian (89.9%), Emirati (80.1%), and Taiwanese (79.3%) populations [14, 21, 30]. However, lower proportions were reported in Saudi (70.6%), Southern Chinese (68.1%), Greek (56.1%), and Polish (59.5%) populations [13, 19, 31, 32].

The present study showed that 5.4% of the studied Yemenis’ MFMs had five root canals. This is in accordance with that reported in the Taiwanese population [21]. However, considerably lower incidences of five canals were reported in Saudi, Polish, Korean, and Chinese populations [13, 17, 31, 32]. On the other hand, the highest reported prevalence was in the Iranian population (11.4%) [14].

Our analysis for the mesiobuccal root of examined MFMs showed that 17.7% had single-canal configuration (Type I), while 82.3% showed 2-canal configurations [Type II (25%), III (23.1), IV (11.3% and VC (14.5%)]. The prevalence of MB2 occurrence in the three-rooted MFMS of the studied Yemeni population was as high as 82.3% which is in accordance with the reported prevalence in Iranian, Taiwanese, and Egyptian populations (86%, 79.2%, and 76.4%, respectively) [14, 15, 21]. However, a lower prevalence has been reported in Korean (63.59%) [17], Thailand (63.3%) [33], Russian (59.8%) [16], Polish (59.5) [31], Greek (53.2%) [19], and Brazilian (42.63%) [34] populations. The lowest reported incidence of MB2 was in the Chinese population [35]. However, higher MB2 prevalence among Chinese was reported in other studies [10, 36]. Although all the aforementioned studies had utilized the same evaluation method (the in vivo CBCT method), a highly variable MB2 prevalence had been reported. This may be attributed to several factors including differences in ethnic origin, sample size, and age range of the studied populations.

The current study showed that the majority (67%) of the MB2 canals join with the first mesiobuccal canal (MB1) and share a single apical foramen. This is close to the finding of Zheng et al. in the Chinese population and Nikoloudaki et al. in the Greek population (79.20% and 80.9%, respectively) [11, 19]. In addition, this finding also agreed with that of Vertucci's study which revealed that 82% of the additional canals in the MB roots merged into one single foramen [5].

Regarding the canal morphology, our findings revealed that single canal configuration (Type I) was more common in the palatal (96%) and distobuccal roots 99.5), respectively. These findings are consistent with several studies carried out in various populations, where the percentage of MFMs with a single canal ranged from 98 to 100% [9, 11, 17, 37, 38].

Although the difference was not significant statistically, the MB2 was found more frequently in MFMs of males (85.8%) than those of females (79.5%) (*P* = 0.07). These findings are consistent with those reported in Chinese, Egyptian, North American, Turkish, and Iranian populations [11, 14, 15, 18, 37]. On the other hand, Kim et al., Sert & Bayirli, Katarzyna & Pawlicka and Ratanajirasut et al. reported a significant male predominance in the MB2 prevalence in MFMs among Korean, Turkish, Polish, and Thai populations, respectively [17, 31, 33, 39].

For the first time in a Yemeni population, the current study examines the root and canal anatomy of MFMs. To some extent, this study will offer a theoretical framework for endodontic therapy. The size of the sample and the methodology of the experiment had a significant impact on the findings of anatomical shapes of root canals. There are a few issues, though, that need to be resolved. This study was cross-sectional, hence the sample size needed to be bigger. Additionally, as the readings may be impacted by the scans' voxel and field sizes, this study is retrospective. The prevalence of these variations in the Yemeni population may be more accurately estimated by multicenter research with bigger sample sizes. The results might have been impacted by the fact that the CBCT employed in this investigation had a lesser spatial resolution than micro- and nano-CT.

## Conclusions

Under the limitations of the current study, it can be concluded that the root canal anatomy is unquestionably influenced by the patient's race. The MFMs' root canal morphology indicated notable variations among a Yemeni population. The MFMs of most of the Yemeni participants in this study had three roots and four canals. The additional fourth canal was mainly located in the mesiobuccal root of examined MFMs. Our findings highlight the significance of looking for and applying cutting-edge approaches to find the MB2 canals in permanent MFMs since they show a high incidence of MB2 (82.3%). Males had a higher proportion than females. Dentists can detect all canals more efficiently if they are aware of the anatomical peculiarities of the teeth beforehand.

## Data Availability

The datasets generated and/or analyzed during the present study are not publicly available as ethics approval was granted on the basis that only the researchers involved in the study could access the identified data but are available and accessible from the corresponding author on reasonable request.

## Abbreviations

MFMs: Maxillary First Molars
3D: Three dimensions
FOV: Fields of view
CBCT: Cone beam computed tomography
MBR: Mesiobuccal root
DBR: Distobuccal root
PR: Palatal root
MB2: Second mesiobuccal canal
DB2: Second distobuccal canal
